# Supplementary material for: Single olfactory receptors set odor detection thresholds
Source: Nat Commun. 2018 Jul 23;9:2887. doi: 10.1038/s41467-018-05129-0 (PMC6056506; doi:10.1038/s41467-018-05129-0)
Supplement: Supplementary file 1 — Supplementary Information [file 41467_2018_5129_MOESM1_ESM.pdf]

Supplementary Information

Single olfactory receptors set odor detection thresholds

Dewan et al.

Correspondence
Thomas Bozza
Department of Neurobiology
Northwestern University
2205 Tech Drive
Hogan 2-160
Evanston, IL 60208
(847) 467-2870
bozza@northwestern.edu

Supplementary Figures

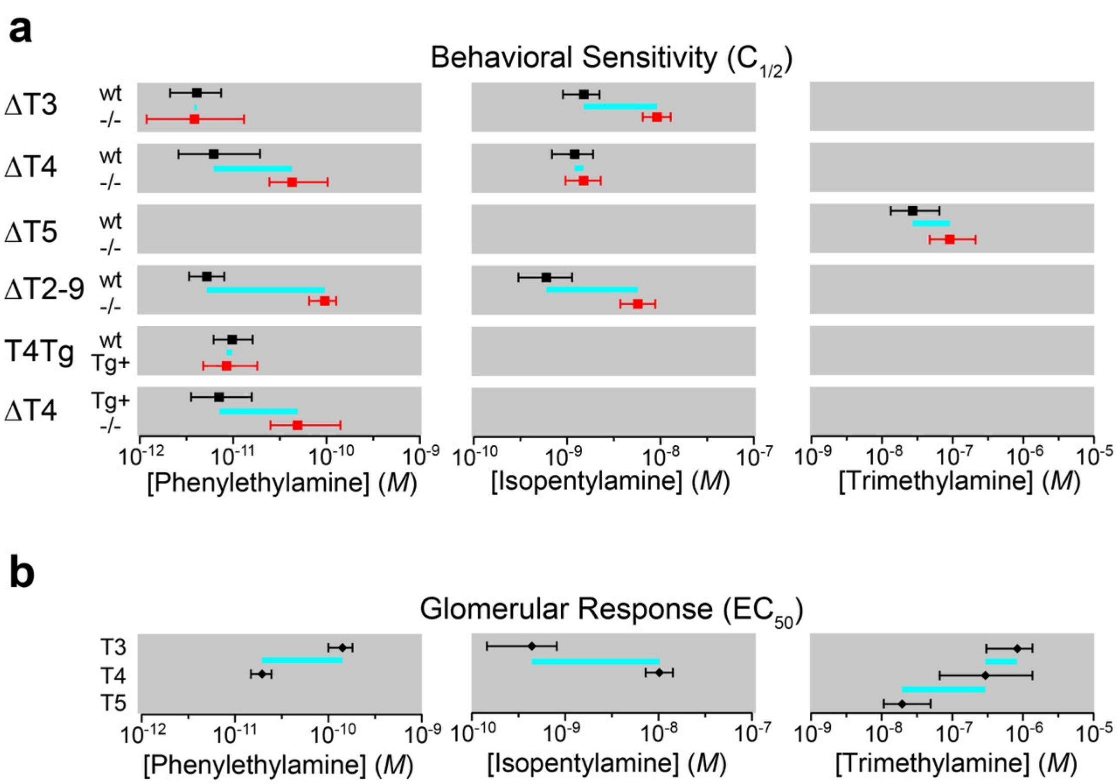

Supplementary Figure 1. Summary of amine sensitivities observed in all experiments. **(a)** Summary of behavioral sensitivities ($C_{1/2}$ values) across 6 gene targeted mutant strains (red) and corresponding wild-type littermate controls or genetic rescue (black). The absolute sensitivity of each mouse strain can vary due to strain dependent variability in task performance. Thus, the most valid comparisons that can be made are within a given strain. The magnitude of sensitivity shifts within strains (mutant compared with corresponding wild-type) are marked by cyan lines. **(b)** Summary of receptor (glomerular) sensitivities (EC_{50} values) from in vivo calcium imaging of TAAR3, TAAR4 and TAAR5 glomeruli. Differences in sensitivity among receptors is indicated by cyan lines. Plots show either mean $C_{1/2}$+/- 95% CI or mean EC_{50} +/- 95% CI (n=6-10

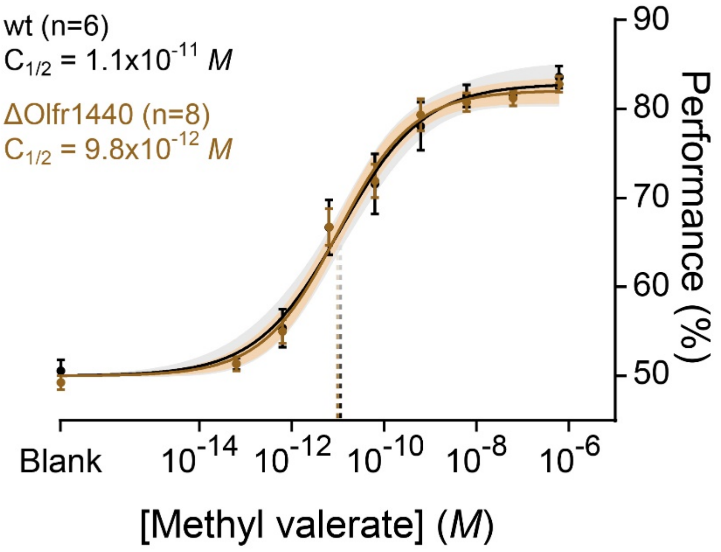

Supplementary Figure 2. Deleting Olfr1440 did not alter sensitivity to the control odor methyl valerate. Psychometric curves for ΔOlfr1440 (tan) and wild-type littermate controls (black). Plot show mean +/- SE with a shaded 95% confidence interval. Curves were fitted via a non-linear regression using a variable slope model with the Hill equation. Behavioral sensitivity is defined as the odor concentration at half-maximal behavioral performance ($C_{1/2}$) during a head-fixed Go No-Go conditioned assay. *wt* $C_{1/2} = 1.1 \times 10^{-11}$ *M* (95% CI = 4.6-31.2 x 10^{-12} *M*); *ΔOlfr1440* $C_{1/2} = 9.8 \times 10^{-12}$ *M* (95% CI 5.7-17.4 x 10^{-12} *M*); p=0.81; F=0.06, Sum-of-squares F-test.

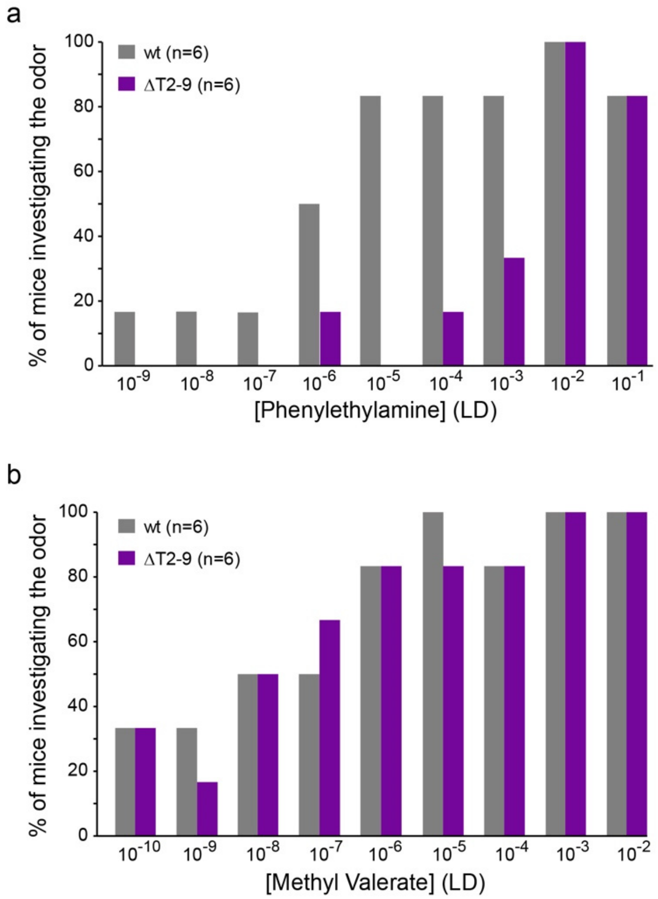

Supplementary Figure 3. Assessment of odor investigation in TAAR cluster deletion mice. Removing the olfactory TAARs causes a pronounced investigation deficit for phenylethylamine but not methyl valerate. Vertical axis represents the percentage of animals that found the odor source (glass capillary dipped in diluted odorant) within 3 minutes. Investigation was defined as active sniffing in close proximity to the odor source. (**a-b**) Percentage of wild-type (dark gray) and cluster deletion mice (purple) that investigated each serial dilution of phenylethylamine (top) and methyl valerate (bottom).
